# Long-Term Survival, Quality of Life, and Psychosocial Outcomes in Advanced Melanoma Patients Treated with Immune Checkpoint Inhibitors

**DOI:** 10.1155/2019/5269062

**Published:** 2019-04-28

**Authors:** Anne Rogiers, Annelies Boekhout, Julia K. Schwarze, Gil Awada, Christian U. Blank, Bart Neyns

**Affiliations:** ^1^Department of Psychiatry, CHU Brugmann, 1020 Brussels, Belgium; ^2^Netherlands Cancer Institute, 1066 CX Amsterdam, Netherlands; ^3^Department of Medical Oncology, UZ Brussels, 1090 Brussels, Belgium

## Abstract

Immune checkpoint inhibitors have become a standard of care option for the treatment of patients with advanced melanoma. Since the approval of the first immune checkpoint (CTLA-4) inhibitor ipilimumab in 2011 and programmed death-1 (PD-1) blocking monoclonal antibodies pembrolizumab and nivolumab thereafter, an increasing proportion of patients with unresectable advanced melanoma achieved long-term overall survival. Little is known about the psychosocial wellbeing, neurocognitive function, and quality of life (QOL) of these survivors. Knowledge about the long term side-effects of these novel treatments is scarce as long-term survivorship is a novel issue in the field of immunotherapy. The purpose of this review is to summarize our current knowledge regarding the survival and safety results of pivotal clinical trials in the field of advanced melanoma and to highlight potential long-term consequences that are likely to impact psychosocial wellbeing, neurocognitive functioning, and QOL. The issues raised substantiate the need for clinical investigation of these issues with the aim of optimizing comprehensive health care for advanced melanoma survivors.

## 1. Introduction

Up to 2010, no medical therapy investigated in a randomized clinical trial had shown to significantly improve overall survival (OS) for patients with unresectable advanced melanoma [1]. Less than half of all patients diagnosed with metastatic melanoma (AJCC stage IV) survived for more than 1 year and only 20% of all patients were alive after 3 years. However, prior to the development of the currently available life-prolonging medical therapies, a small percentage of patients with advanced melanoma experienced long-term survival for more than 5 years. The characteristics of this small subpopulation have never been fully elucidated. Patients with natural indolent evolution of metastatic disease and cases suspect of “spontaneous immune mediated remission” (often coincident with the development of vitiligo) are likely to have contributed to this historical “tail of the survival curve” for stage IV melanoma. In addition, complete resection of oligometastatic stage IV disease can occasionally provide durable remission in a small proportion of patients, but identifying these patients prospectively on objective clinical or histopathological characteristics has not been achieved and requires further investigation. Finally, durable remissions and long-term survival following conventional cytotoxic chemotherapy (e.g., dacarbazine, temozolomide) have also been reported in exceptional cases, most often after a complete response (CR) had occurred [2].

In the 1980s, it was established that a small percentage of patients with favorable baseline characteristics who were treated with high-dose interleukin-2 (IL-2) could achieve a durable complete remission. In a comprehensive review of the outcome of 270 patients with unresectable melanoma (8 clinical trials conducted between 1985 and 1993), receiving IL-2 administered at a high dose resulted in a complete response (CR) in 6% and a partial response (PR) in an additional 10% of patients. A CR seemed a prerequisite for durable progression-free survival (PFS) as the median response duration in patients obtaining a PR was limited to 5.9 months. These IL-2 treatment regimens were associated with substantial toxicity with grade 5 adverse events (AE) occurring in 2% of patients. The two baseline predictive factors for response to high-dose IL-2 therapy were the performance status and whether patients had received prior systemic therapy. Combination regimens of IL-2, interferon-*α* (IFN-*α*), and cisplatin-based combination chemotherapies, while showing high overall response rates with some durable remissions, failed to significantly improve survival rates for patients with advanced melanoma and were subsequently abandoned [3].

Since 2010 effective systemic therapies have become available that improved OS of patients with advanced melanoma. Effective new therapies target the T-cell inhibitory immune checkpoint receptors (including the cytotoxic T-lymphocyte-associated antigen 4 (CTLA-4) and Programmed Death 1 (PD-1) receptors on lymphocytes), or the MAPK-signaling pathway in patients with* BRAF*^*V600*^ mutant melanoma, as well as more recently talimogene laherparepvec (T-VEC, the first approved oncolytic virotherapy for cancer offering a survival benefit in patients with stage IV-M1a). Since 2010, all phase III studies conducted with these new agents have reached their primary endpoint, demonstrating improved OS and thereby revolutionizing the treatment options for patients with unresectable advanced melanoma.

## 2. Ipilimumab

**Figure 1:**
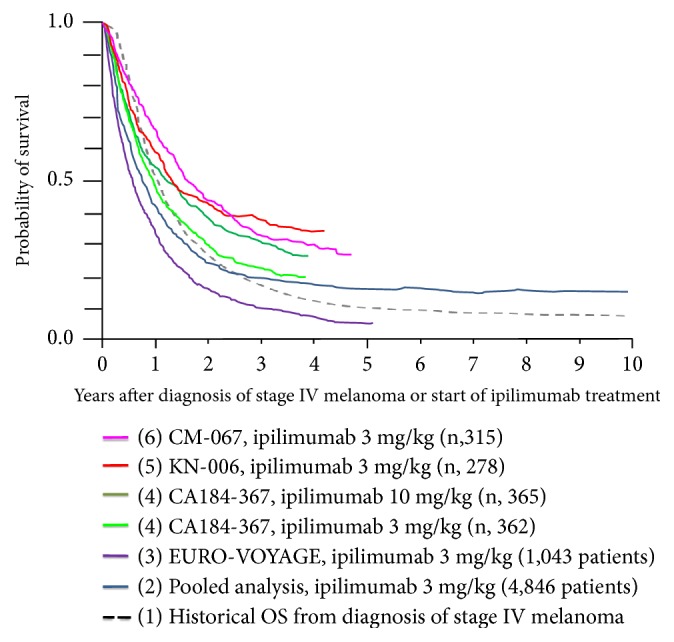
*Overlay of Kaplan–Meier curves indicating the probability for overall survival (OS) for patients treated with ipilimumab as first line of immunotherapy*, representing (1) the historical probability for OS for patients diagnosed with stage IV melanoma prior to the availability of life-prolonging medical treatment options (dashed black line) [1]; (2) a pooled OS analysis including individual patient survival data from 1,861 patients with metastatic melanoma from 12 clinical investigations of ipilimumab and 2,985 patients with metastatic melanoma from a US ipilimumab EAP (total n = 4,846) (dark blue line) [9]; (3) interim results from EURO-VOYAGE, a multicenter, observational, retrospective study of 1043 patients with advanced melanoma who participated in the EU ipilimumab EAP (purple line) [69]; (4) intention-to-treat population (365+362 patients) of the CA184-367 study comparing ipilimumab at 10 mg/kg (dark green line) to 3 mg/kg dosing level (light green line) [8]; (5) intention-to-treat population (278 patients) on the ipilimumab arm from the Keynote-006 trial (red line) [13]; (6) intention-to-treat population (315 patients) on the ipilimumab arm from the Checkmate-067 trial (pink line) [72].

The first systemic treatment ever to significantly improve OS for patients with unresectable advanced melanoma was the CTLA-4 blocking monoclonal antibody (mAb) ipilimumab. This drug was approved in 2011 based on the study outcomes of two randomized phase III trials. The first trial, CA184-002, compared ipilimumab (administered at a dose of 3 mg/kg intravenously [IV] every 3 weeks for a total of four consecutive doses) to a gp100 vaccine or the combination of both in HLA-2 positive patients with pretreated advanced melanoma [4]. For patients, with stable disease after at least 12 weeks of treatment, and who subsequently were diagnosed with progression of disease, reinduction with ipilimumab was allowed. The objective tumor responses according to the Response Criteria in Solid Tumors (RECIST) criteria ranged from 5.7% to 11.0% in the ipilimumab treatment arms. The median OS was improved to 10.0 months for the ipilimumab monotherapy-arm as compared to 6.4 months for the peptide vaccine-alone arm (HR 0.68; p < 0.001) (Figure 1). Combination of ipilimumab with the gp-100 vaccine provided no benefit over ipilimumab alone (Table 1).

In a second pivotal phase III study (CA184-024), ipilimumab (administered at a dose of 10 mg/kg every 3 weeks for a total of four consecutive doses and subsequently once every 12 weeks) was combined with dacarbazine chemotherapy (850 mg/m^2^) and compared with dacarbazine plus placebo. Median OS was improved for ipilimumab plus dacarbazine (11.2 months) as compared to dacarbazine alone (9.1 months; HR 0.72; p < 0.001) (Table 1, Figure 1). The co-administration of ipilimumab with dacarbazine significantly increased the incidence of grade 3 or 4 toxicity hepatic toxicity (grade 3 or 4 AEs occurred in 56.3% of patients treated with ipilimumab plus dacarbazine, as compared with 27.5% treated with dacarbazine and placebo) and hepatotoxicity in particular (grade 3 or 4 elevations in liver-function values noted in 17.4 to 20.7% of the patients) [5].

Additional evidence for the long-term beneficial survival effect from ipilimumab came from a large randomized phase II trial in pretreated patients comparing the 0.3, 3, and 10 mg/kg dose levels, indicating a dose-dependent outcome in terms of objective tumor response rate and survival, but also a dose-dependent increase in toxicity [65]. In 2011, ipilimumab received approval by the competent authorities in Europe, the US, and Australia for the treatment of advanced melanoma at a dose of 3 mg/kg administered every 3 weeks for a total of four consecutive doses. The label did not include a reference to the possibility of retreating patients who responded to the initial four doses. Although only a small proportion of the CA184-002 study population was retreated at the time of first progression following an initial favorable response to ipilimumab, this may have contributed to the long-term (≥3 year) survival results on ipilimumab monotherapy [66] (Figure 1).

Novel features of ipilimumab therapy included an increased potential for long-term survival benefit in a small proportion of patients, the occurrence of new adverse events (AEs), the so-called “immune-related AEs” (irAE), and the atypical kinetics of treatment response [67, 68]. A consistent finding across these clinical trials investigating ipilimumab was the absence of a measurable impact on OS in the first 3 to 4 months of treatment. With longer followup a moderate improvement of the median OS outcome became apparent, and the long-term probability for survival after 3 years or longer (the so-called “tails of the survival curves”) was not reconverting, indicative of the fact that 10-15% of the ipilimumab treated population derived a highly durable survival benefit as compared to the control population. Mature survival data were reported in an updated report of survival rate of the CA184-014 trial and a pooled analysis of 1861 patients from 10 prospective and two retrospective studies: 5y-OS rate was 18.2% (95% CI, 13.6% to 23.4%) for patients treated with ipilimumab plus dacarbazine versus 8.8% (95% CI, 5.7% to 12.8%) for patients treated with placebo plus dacarbazine (P = .002) (Table 1). An “inflexion-point” on the curve followed by a plateau in the survival curve began at approximately 3 years [6] (Figure 1).

These findings were confirmed in a pooled analysis including 1861 patients from ten prospective and two retrospective studies, including a majority of patients receiving ipilimumab according to the 3 mg/kg (n = 965) or 10 mg/kg (n = 706) dose levels (Table 1). Twenty-two percent of the patients were alive at 3 years, and a plateau on the survival curve became apparent 3 years after the start of treatment [9]. A second analysis of OS data with a total of n = 4,846 patients (including an additional 2,985 patients from an expanded access program) further confirmed a survival plateau at 21% from 3 years on (Figure 1).

Following approval of ipilimumab in 2011, a phase III trial (CA184-367), was conducted to address the unresolved question regarding the optimal dosing of ipilimumab (3 vs. 10 mg/kg); 727 patients without prior exposure to BRAF or PD-1 inhibitors were randomly assigned (1:1) to ipilimumab with either dose level [8]. The median number of doses of ipilimumab administered was four in each arm, with retreatment being pursued in a minority of patients (6% and 9% of patients in the 10 and 3 mg/kg arms, respectively). The median OS was superior for patients treated on the 10 mg/kg arm (15.7 versus 11.5 months; HR 0.84; p = 0.04) (Table 1). No difference in the probability for survival was evident during the first 6 months of followup. Thereafter the curves separated and a distinct 2- and 3-year survival rate was observed between both dose levels of ipilimumab (Figure 1). Treatment-related AEs in the 10 mg/kg arm were more frequent as compared to the 3 mg/kg arm (79% all-grade and 34% grade 3 to 5 AEs, as compared with 54% and 14%).

More recently, the effectiveness of ipilimumab was examined in a systematic retrospective analysis of 1034 patients with advanced melanoma who were included in a European Expanded Access Program (EURO-VOYAGE). A median OS of 6.8 months was found and the 3- and 4-years OS rates were, respectively, 10.9 and 8% and thus were apparently lower than what had been reported before (Table 1) [69]. These results indicate that the level of the “tail of the survival curve” remains dependent on the baseline characteristics of the investigated population, with an important role for baseline covariables as determinants for durable survival for patients treated with ipilimumab (Figure 1) [7, 70, 71].

## 3. Anti-PD-1 Therapies

Since 2015, ipilimumab has been replaced as the preferred first choice immunotherapy for advanced melanoma by PD-1 blocking mAb. Pembrolizumab and nivolumab were approved in the EU, US, and Australia as first-line immunotherapy for advanced melanoma based on phase III trials demonstrating a significant improvement of both PFS and OS as compared to ipilimumab [74, 75]. Notwithstanding the relative short followup of up to 3-4 years for these study populations, superior survival rates have been reported at every land-mark analysis [72, 76]. Moreover, followup of patient populations treated on phase I trials with nivolumab and pembrolizumab have also demonstrated the potential for durable survival gains after up to 5 years of followup [10, 11]. Anti-PD1 therapies are associated with a lower incidence of immune-related AEs as compared to ipilimumab [12].

### 3.1. Pembrolizumab

**Figure 2:**
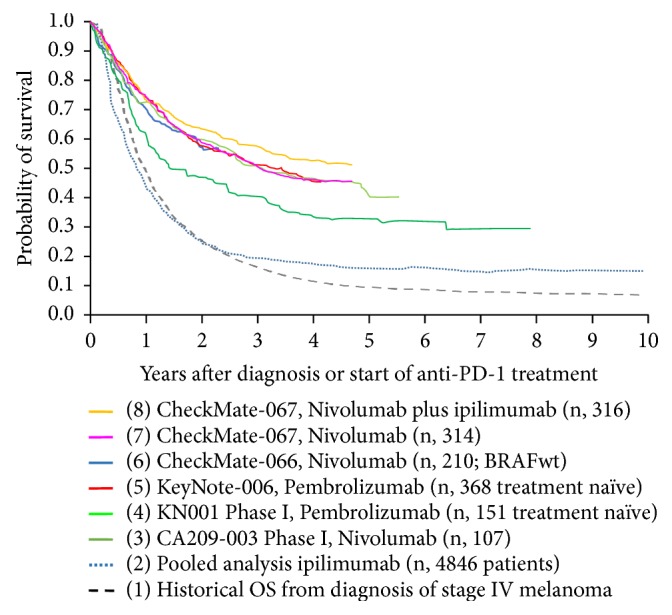
*Overlay of Kaplan–Meier curves indicating the probability for OS (OS) for advanced melanoma patients treated with anti-PD1 as first-line immunotherapy*, representing (1) the historical probability for OS for patients diagnosed with stage IV melanoma prior to the availability of life-prolonging medical treatment options (dashed black line) [1]; (2) a pooled OS analysis including individual patient survival data from 1,861 patients with metastatic melanoma from 12 clinical investigations of ipilimumab and 2,985 patients with metastatic melanoma from a US ipilimumab EAP (total n = 4,846) (blue line) [9]; (3) CA209-003 phase I clinical trial with nivolumab for pretreated advanced melanoma patients (dark green line) [11]; (4) treatment naïve patients (n: 151) treated in the Keynote-001 clinical trial with pembrolizumab (light green line) [10]; (5) treatment naïve patients (n, 368) from the Keynote-006 trial (red line) [13]; (6) nivolumab treated patients with BRAF V600 wild-type melanoma (n, 210) from the Checkmate-066 trial (blue line) [73]; (7) nivolumab monotherapy treated patients (n, 314) from the CheckMate-067 trial (pink line) [72]; (8) nivolumab plus ipilimumab treated patients (n, 316) from the CheckMate-067 trial (orange line) [72].

In the KEYNOTE-006 phase III trial, patients with unresectable stage III or IV melanoma had been randomly assigned (1:1:1) to one of two dose regimens of pembrolizumab (10 mg/kg every 2 or 3 weeks) or one regimen of ipilimumab (3 mg/kg every 3 weeks for a total of 4 consecutive doses) [13] (Table 1). Pembrolizumab treatment was continued for a maximum duration of 2 years. After a median followup of 22.9 months, median OS was not reached in either pembrolizumab group and was 16.0 months with ipilimumab (hazard ratio [HR] 0.68, 95% CI 0.53-0.87 for pembrolizumab every 2 weeks vs. ipilimumab p=0.0009; and 0.68, 0.53-0.86 for pembrolizumab every 3 weeks vs. ipilimumab; p=0.0008) with a 24-month OS rate of 55% for pembrolizumab treated patients and 43% in the ipilimumab group. The 33-month PFS-rate was 31 vs. 14% and OS-rate 50 vs. 39% for the pooled pembrolizumab arms vs. the ipilimumab group. After a median followup of 45.9 months (range: 0.3-50.0) the 4-year OS rates were 42% in the pooled pembrolizumab groups and 34% in the ipilimumab group (Figure 2). One hundred and three patients (19%) received the maximum duration of 2 years of pembrolizumab treatment and only 14% of the patients experienced progressive disease (median followup of 20.3 months) [13].

In an open-label phase 1b clinical trial (KEYNOTE-001) patients received pembrolizumab 2 mg/kg or 10 mg/kg every 3 weeks or 10 mg/kg every 2 weeks until disease progression or intolerable toxicity. The median OS was 23.8 months in all 655 patients, with 3-year and 4-year survival estimates of 42% and 37% (Table 1). In the 152 treatment-naïve patients, the 3-year and 4-year survival estimates were 51% and 48%, respectively (Table 1). Recently the updated 5 years overall survival results have been published and an OS of 34% in all patients and an OS of 41% in treatment-naïve patients were found (Figure 2) [10, 76, 77].

### 3.2. Nivolumab

Similar survival outcome has been observed in another double-blind, phase 3 study, investigating nivolumab alone or nivolumab plus ipilimumab versus ipilimumab alone as first line therapy in 945 previously untreated patients with unresectable stage III or IV melanoma (Checkmate-067) [14]. Both nivolumab containing treatment arms significantly improved both PFS and OS as compared to ipilimumab and a superior PFS was obtained in the combination arm of nivolumab and ipilimumab (Table 1). However, treatment-related AEs of grade 3 or 4 occurred more frequently with upfront combination of nivolumab and ipilimumab (22.4% of the patients in the nivolumab monotherapy arm, 59.1% of those in the combination arm, and 27.7% of those in the ipilimumab monotherapy arm). After a minimum followup of 48 months, the median OS had not been reached in the combination group and was 36.9 months in the nivolumab monotherapy group, as compared with 19.9 months in the ipilimumab monotherapy group (hazard ratio for death with nivolumab plus ipilimumab vs. ipilimumab, 0.54 [P < 0.001]; hazard ratio for death with nivolumab vs. ipilimumab, 0.65 [P<0.001]). The OS rate at 4 years was 53% in the nivolumab-plus-ipilimumab and 46% in the nivolumab monotherapy arm, as compared with 30% in the ipilimumab monotherapy arm (Figure 2). The two groups including nivolumab had significantly longer survival compared to the ipilimumab group. In a descriptive analysis, the hazard ratio for death with nivolumab plus ipilimumab versus nivolumab monotherapy was not statistically significant (hazard ratio for death was 0.84 with a 95% CI, 0.67 to 1.05).

Comparable to patients treated with pembrolizumab, the hazard ratio for progression of disease decreased with time and the rate of PFS at 4 years was 37% in the nivolumab-plus-ipilimumab group and 31% in the nivolumab monotherapy arm, as compared with 9% in the ipilimumab monotherapy arm. In a descriptive analysis, the hazard ratio for progression or death was 0.79 (95% CI, 0.65 to 0.97) with nivolumab plus ipilimumab versus nivolumab indicating the potential for a stable survival plateau above 30% in both nivolumab treatment arms (Figure 2).

Long-term prediction of OS-rates in patients with advanced melanoma treated with anti-PD-1 mAb is currently only available for pretreated patient populations who participated in phase I clinical trial programs. The available data nevertheless are indicative that the OS probability curve is likely to reach a plateau. Thirty-four percent of patients treated with nivolumab in a phase I trial (CA209-003) were alive 5 years after initiating study treatment [49].

### 3.3. Real-World Outcome Data on Anti-PD-1 Therapy

In a poster presented at the SMR 2017 annual meeting real-world outcome data were reported on 189 advanced melanoma patients discontinuing anti-PD-1 treatment (pembrolizumab or nivolumab) in the absence of PD or treatment limiting toxicity [15]. Data were collected at 14 hospitals across Europe and Australia. Short-term outcome of patients that stopped therapy in absence of progression of disease or treatment limiting toxicity was encouraging, with a low-risk for PD (4% after a median FU of 35 weeks). Reintroduction of a PD-1-inhibitor in patients who progressed after discontinuation (n= 9 patients) indicated the potential for renewed antitumor activity. Additional reports, in line with these results on real-world outcome data, were recently reported by additional groups [16–19].

## 4. Long-Term Immune Related Adverse Events

The side effects of immune checkpoint blockade are often referred to as immune-related adverse events (irAE). The most common irAE occur in skin, liver, and gastrointestinal, pulmonary, and endocrine organs but autoimmune diabetes and cardiovascular, renal, and musculoskeletal side effects are also reported [20, 21]. Most cutaneous, gastrointestinal, and hepatic AEs occurred within two months, whereas endocrine, pulmonary, and renal side effects appeared after 9 weeks [68]. Early diagnosis and treatment are believed to be important in mitigating the severity of irAEs [22]. Most of these irAE are reversible after treatment interruption and/or steroid therapy; however, the endocrine irAE (most commonly hypophysitis and thyroiditis) may necessitate life-long hormonal substitution [23, 24]. One study on 15 patients diagnosed with autoimmune hypophysitis induced by ipilimumab treatment reported that all patients had at least one hormonal defect at diagnosis [25]. In all patients clinical symptoms improved in the first month after starting glucocorticoid therapy. At the end of followup (median 33.6 months, range 7-53.5), 13 (86.6%) required long-term hormonal replacement with corticotropic deficiency persisting in all patients suffering from hypocorticism. No prospective study results are currently available on the long-term (>3 yrs) consequences of irAEs.

The high incidence of irAE observed with the combination of nivolumab (1mg/kg) and ipilimumab (3 mg/kg) has prompted the investigation of nivolumab or pembrolizumab combined with ipilimumab at a lower dose level of 1 mg/kg every 3 weeks [26, 27]. The CheckMate 511 study demonstrated a significantly lower incidence of treatment-related grade 3-5 AEs. However, longer followup is needed to address the long-term OS outcome as for ipilimumab in monotherapy, long-term OS is dose dependent.

## 5. Health Related Quality of Life in Melanoma Survivors

To date patient reported global health related quality of life (HRQOL) is measured using several valid instruments to assess different dimensions of HRQOL, such as psychological, social, physical, and spiritual aspects. HRQOL instruments can be generic, cancer specific, or cancer disease specific and measure only one or several dimensions. Commonly used scales to assess the global HRQOL in cancer patients include the European Organization for Research and Treatment of Cancer Quality of life Questionnaire (EORTC-QLQ-C30), the Impact of Cancer questionnaire (IOC), and the Generic Functional Assessment of cancer therapy (FACT-G) for which an additional melanoma scale was validated, the FACT melanoma (FACT-M) [28–31]. In the field of survivorship the EORTC-QOL survivorship questionnaire is currently in validation process [32].

Results from three randomized controlled trials (MDX010-20, KEYNOTE-002, and CheckMate 067) suggest that ipilimumab, nivolumab, and pembrolizumab, as a monotherapy, and the combination therapy of nivolumab plus ipilimumab or ipilimumab plus gp100 vaccine are well tolerated and either improve or maintain HRQOL as assessed with the EORTC QLQ-C30 scale, during the treatment induction phase [33–35]. However, there might be an underestimation of the influence of these treatments on the HRQOL because of low patient numbers in the later weeks of all studies due to disease progression, death, and AEs. In the KEYNOTE-006 it has been observed that the HRQOL assessed with the EORTC QLQ-C30 scale, in patients treated with pembrolizumab, was better maintained as compared to ipilimumab in patients with ipilimumab naïve advanced melanoma (Table 2) [34].

In a systematic review of 7 studies (4246 patients; 6 cross-sectional [36–44], and 1 prospective study [45]), it was found that determinants of lower HRQOL (either psychological, physical, or global) were marital status, age, sex, poor social support, melanoma severity at diagnosis, and comorbidities (Table 3) [46]. Dieng et al. found that HRQOL, measured with the Functional Assessment of Cancer Therapy (FACT-M), was correlated with fear of recurrence of disease in patients with metastatic melanoma (Table 3) [47]. A more comprehensive understanding of HRQOL can improve patient centered care in melanoma patients. In addition HRQOL assessment can be used as outcome measure for cancer research and help socioeconomic decision making. Therefore international consensus on how to assess HRQOL is mandatory, as well as the development and validation of melanoma specific assessment tools [46, 48]. In Table 4 an overview is given of the characteristics of the questionnaires used in the referenced trials.

## 6. Psychosocial Outcomes in Melanoma Survivors

With increasing numbers of advanced melanoma patients becoming long-term cancer survivors, even after discontinuing therapy, the issue of melanoma survivorship care becomes of relevance to more patients than ever before. Cancer survivorship has been extensively studied in other cancer indications [49]. In these studies, cancer survivors have been reported to suffer from mental and physical symptoms, fatigue, and neurocognitive dysfunction persisting after physical recovery from their disease. These mental and neurocognitive symptoms are associated with important psychosocial consequences such as delayed return to work, impaired family relationships, and reduced quality of life (QOL) [77, 78].

Only a few studies are focusing on psychosocial outcome in melanoma survivors, with all of them showing diminished wellbeing, high levels of distress, and fear for recurrence (Table 3) [36, 43]. Nevertheless, results of these studies are limited as they are all survey-based and mainly include patients with early stage melanoma and in a lesser extent nonmetastatic disease treated with adjuvant therapy. The following risk factors have been described to be related to higher distress in early stage melanoma patients: female gender, younger age, negative appraisal, and negative coping strategies (Table 3) [63]. Higher distress and fear of recurrence might be related to the necessity of continued self-examination, dermatological controls, and reduced sun exposure [37, 79]. Moreover, higher anxiety levels and fear for recurrence are associated with avoidance behavior in relation to dermatological controls [80]. The traumatic course of metastatic melanoma may also contribute to more difficult coping mechanism as compared to other cancer indications [46]. In accordance with these findings, Dieng explored the usefulness of psychoeducational intervention in patients diagnosed with stages 1-2 melanoma and found a substantial benefit compared to the patients who received standard of care [81].

Currently no data are available on the potential long-term emotional, physical and cognitive side effects of immune checkpoint inhibitors in patients with metastatic melanoma.

Moreover, an important subgroup of patients with brain metastasis is becoming survivors, which makes it imperative to study potential effects on neurocognitive functioning, especially because survivors who have previously been irradiated for brain metastases are at increased risk for focal postradiation necrosis of the brain [82]. Efforts to further comprehensively address these psychosocial, neurocognitive, and HRQOL issues are ongoing at present at our department. Preliminary observations indicate that a substantial fraction of these patients experience diminished HRQOL, persisting fatigue, severe emotional disturbances, and neurocognitive complaints [83, 84]. A multicentric study addressing HRQOL in long-term survivors following treatment with ipilimumab is currently ongoing in The Netherlands and Belgium.

In conclusion prospective investigation of the potential psychosocial, neurocognitive, and HRQOL issues is needed, in order to identify the care needs of advanced melanoma survivors. Optimizing patients' subjective wellbeing could potentially reduce the emotional, physical, and socioeconomic consequences of this devastating disease.

## Figures and Tables

**Table 1:** Key features of referenced clinical trials with immune checkpoint inhibitors for advanced melanoma.

Name clinical trial Phase	Number of patients	Treatment plan	Primary endpoint	Median OS (95% CI)	Median progression-free survival PFS (95% CI)	Overall survival rates OS
CA184-002 Phase III [4]	676	G1:IPI 3 mg/kg + gp100 G2: IPI G3: gp100 Dose: Every 3 weeks for four cycles Mode: IV	OS	IPI + gp100: 10.0 mos. (8.5-11.5) IPI: 10.1 mos. (8.0-13.8) gp100: 6.4 mos. (5.5-8.7)	IPI + gp100: 2.76 mos. (2.73-2.79) IPI: 2.86 mos. (2.76-3.02) gp100, 2.76 mos. (2.73-2.83)	IPI + gp100, IPI, gp100: 1 yr.: 43.6% vs 45.6% vs 25.3% 2 yrs.: 21.6%, 23.5%, 13.7%

CA184-024 Phase III [5, 6]	502	G1: IPI 10 mg/kg + dacarbazine 850mg/m^2^ G2: dacarbazine + placebo Dose: weeks 1, 4, 7, and 10, followed by dacarbazine monotherapy every 3 weeks until week 22. Mode: IV	OS	IPI + dacarbazine: 11.2 mos. (9.4-13.6) Dacarbazine + placebo: 9.1 mos. (7.8-10.5)	Median values for PFS were similar in the two groups at week 12	IPI + dacarbazine, dacarbazine: 1 yr.: 47.3% vs. 36.3% 2 yrs.: 28.5% vs. 17.9% 3 yrs.: 20.8% vs. 12.2% 5 yrs.: 18.2% vs. 8.8%

Expanded access program EURO-VOYAGE [7]	1034	IPI 3 mg/kg	OS	6.8 mos. (6.1-7.4)	Median PFS 2.6 mos. (2.6-2.7)	3 yrs.: 10.9 % 4 yrs.: 8%

CA184-367 III [8]	727	G1: IPI 3 mg/kg G2: IPI 10 mg/kg	OS	IPI 3 mg/kg: 11.5 mos. (9.9-13.3) IPI 10 mg/kg: 15.7 mos. (6-17.8)	IPI 3mg/kg: 2.8 mos. (2.8-2.8); IPI 10 mg/kg, 2.8 mos. (2.8-3.0)	IPI 3 mg/kg, IPI 10 mg/kg: 1 yr.: 47.6% vs. 54.3% 2 yrs.: 31.0% vs. 38.5% 3 yrs.: 23.2% vs. 31.2%

Pooled analysis from Phase II and Phase III [9]	1861	The majority of patients had received IPI 3 mg/kg or 10 mg/kg	OS	11.4 mos. (10.7-12.1)		3 yrs.: 22% for all patients, 26% for treatment-naïve patients and 20% for previously treated patients

KEYNOTE-001 Phase Ib [10–49]	655	PEMBRO 2 mg/kg every 3 weeks, PEMBRO 10 mg/kg every 3 weeks or PEMBRO 10 mg/kg every 2 weeks until disease progression or intolerable toxicity	CR	23.8 mos. (20.2-30.4)	8.3 mos. (5.8-11.1) in all treated patients 16.9 mos. (9.3-35.5) in treatment naïve patients	3 yrs.: 42% in all treated patients; 51% in treatment-naïve patients 4 yrs.: 37% in all treated patients; 48% in treatment-naïve patients 5 yrs.: 34 % in all patients, 41% in treatment naive

KEYNOTE-006 Phase III [13]	834	PEMBRO 10mg/kg every 2 weeks PEMBRO 10 mg/kg every 3 weeks IPI 3 mg/kg every 3 weeks for four cycles	PFS and OS	Median OS was not reached in the resp. PEMBRO arms IPI: 16.0 mos.	PEMBRO every 2 weeks, 5.5 mos. (3.4-6.9); PEMBRO every 3 weeks 4.1 mos. (2.9-6.9); IPI 2.8 mos. (2.8-2.9)	PEMBRO every 2 weeks, PEMBRO every 3 weeks, IPI: 1 yr.: 74.1% vs 68.4% vs 58.2% 2 yrs.: 55% vs. 55% vs. 43%

Checkmate-067 Phase III [14]	945	NIVO 3 mg/kg or NIVO 1mg/kg + IPI 3 mg/kg every 3 weeks for 4 doses followed by NIVO 3 mg/kg every 2 weeks or IPI 3 mg/kg every 3 weeks for 4 doses	PFS	NIVO, 37.6 mos. (29.1 to not reached); NIVO + IPI not reached; IPI 19.9 mos. (16.9-24.6)	NIVO, 6.9 mos. (5.1-9.7); NIVO+IPI, 11.5 mos. (8.7-19.3); IPI, 2.9 mos. (2.8-3.2)	NIVO, NIVO+IPI, IPI: 3 yrs.: 52% vs. 58% vs. 34% 4 yrs.: 46% vs 53% vs 30%

CI, confidence interval; CR, complete response; gp100, glycoprotein 100 peptide vaccine; IPI, ipilimumab; NIVO, nivolumab; OS, overall survival; PEMBRO, pembrolizumab; PFS, progression-free survival; mos., month; yr., year.

**Table 2:** Key features of referenced trials investigating Health Related Quality of life in patients treated with immune-checkpoint inhibitors.

First author	Study design	Study population and AJCC stage	Assessment of quality of life	Sample size	Response rate	Main conclusions on HRQOL
Revicki D. A. et al [33]	Phase III MDX010-20	Stage IIIc/IV pts. during treatment induction	EORTC QLQ-C30 at baseline and week 12	676 pts.: IPI + gp100: N=403 IPI alone: N=137 Gp alone: N=136	Baseline ≥ 95% Week 12: IPI + pg100: 62% IPI alone: 65 % Gp alone: 61%	IPI with or without gp100 does not have significant negative impact on HRQOL during the induction phase compared to gp100 alone.

Petrella T. M. et al [50]	Phase III KEYNOTE-006	Stage IIIc/IV pts. during treatment induction	EORTC QLQ-C30 EQ-5D at baseline and week 12	776 pts.: PEMBRO every 2 w.: N=270 PEMBRO every 3 w.: N=266 IPI 3 mg/kg: N=240	Baseline ≥ 98% Week 12: PEMBRO 2 w: 79% PEMBRO 3 w: 85% IPI: 74%	HRQOL was better maintained with PEMBRO than with IPI in patients with IPI-naive advanced melanoma.

Schadendorf D. et al [35]	Phase III Checkmate-067	Stage IIIc/IV pts. during first 12 months of treatment	EORTC QLQ-C30 EQ-5D at baseline there after resp. w. 1 and 5 of every 6 w. cycle during first 6 mos., and every 6 w. thereafter	945 pts.: NIVO: N=316 NIVO + IPI: N=314 IPI: N=315	Baseline ≥ 89% Week 13: NIVO: 78% NIVO + IPI: 53% IPI: 63%	Results of HRQOL data support the clinical benefit of NIVO monotherapy and NIVO plus IPI combination therapy in pts. with advanced melanoma. Differences in irAE between the 2 groups did not affect HRQOL.

Schadendorf D. et al [34]	Phase III KEYNOTE-002	Stage IIIc/IV pts. during the first 12 weeks	EORTC QLQ-C30	520 pts.: PEMBRO 2 mg/kg: N=176 PEMBRO 10 mg/kg: N=177 Chemotherapy: N=167	Baseline: Week 12 ≥ 93% PEMBRO 2 mg/kg: 69% PEMBRO 10 mg/kg: 75% Chemotherapy: 65%	HRQOL was better maintained with PEMBRO than with chemotherapy, supporting the use of PEMBRO in pts. with IPI-refractory melanoma

gp100, glycoprotein 100 peptide vaccine; IPI, ipilimumab; NIVO, nivolumab; PEMBRO, pembrolizumab; yr., year; w., week; pts., patients; HRQOL: Health Related Quality of Life; SF-36, Short Form 36.

**Table 3:** Key features of the referenced studies with the main findings on psychosocial outcome.

First author	Study design	AJCC stage Time (T) since diagnosis	Questionnaires	Sample size (response rate)	Main findings on HRQOL	Main findings on psychosocial outcome
Beutel M. E. et al [36] Fishbeck S. et al [43]	Cross-sectional Survey	Mainly stage I/II (41% staging was missing) T since diagnosis: 6 - 9 yrs. (70%), ≥ 10 yrs. (30%)	EORTC QLQ-C30 [28] Health Questionnaire Depression (PHQ-9) [51] Multidimensional General Anxiety disorder (GAD-7) [52] Illness specific support Scale (ISSS) [53]	1320 (52%)	Global HRQOL was comparable to general population Lower emotional, cognitive and social functioning and higher symptom burden compared to general population.	Increased depression and anxiety compared to the general population. 36% was in need of psychosocial support. Fear of recurrence of disease caused the highest burden.

Cromwell K.D. et al [45]	Prospective longitudinal study	Stage III T since diagnosis: 0-30 mos.	FACT-M [31] Lymphedema and Breast cancer questionnaire (LBCQ) [54]	277 (71%)	Lymphedema impacts HRQOL.	Lower extremity lymphedema pts. cope less effectively but improve over time Household chores and sleep are most impacted by lymphedema.

Palesh O. et al [37]	Cross-sectional survey	Stage unknown Median T since diagnosis 77 mos, range(0-336)	Non validated electronically administered survey	893 (18%)	_	Melanoma survivors experience continuing anxiety long after treatment. 30% of the pts. reported emotional distress. Long term survivors decreased use of skin protection and frequency of skin screening.

Schubert-Fritze et al [38] Schlesinger Raab A. et al [44]	Cross-sectional survey	Stage I/II T since diagnosis: 2 yrs.	EORTC QLQ-C30 [28] FACT-G [30, 55] Mental Adjustment to Cancer Scale [56]	1085 (61%)	Global HRQOL was comparable with the general population. Number of co-morbidities, age and lymphadenectomy increased the risk for worse global HRQOL, role functioning and worry about the future.	Doctor patient communication was correlated with emotional and social functioning. 42% of the pts. worried about recurrence of disease.

Hamama-Raz Y et al [39, 40]	Cross-sectional survey	Stage I/II T since diagnosis: 5 yrs. (36%) ≥ 5 yrs. (64%)	Mental Health Inventory (MHI) [57] Cognitive Appraisal of Health Scale [58]	400 (75%)	Mean well-being score and mean distress score are similar compared to general population	Subjective factors, such as appraisal of the threat, may be more predictive than medical factors in coping with cancer. Men and women cope differently.

Waldmann et al [41]	Cross-sectional survey	Stage I/II (59%) Stage III (17%) Stage IV (1.9%) T since diagnosis: Q1: 15 mos. Q2: 39 mos.	EORTC QLQ-C30 [28]	762 (59%)	No clinical meaningful differences on global HRQOL between Q1 and Q2.	_

Holterhues C et al [42]	Cross sectional survey	Stage I/II (81%) Stage III (8%) Mean T since diagnosis: 4.6 (2.6) yrs.	Short Form Health Survey (SF-36) [59] Impact of Cancer scale (IOC) [60]	699 (80%)	Medical co-morbidity and female were the main predictors of impaired HRQOL. Impairment of HRQOL seems to be melanoma specific.	Time since diagnosis, tumor stage and co-morbidity were predictors of negative IOC scores. 85 pts. (35%) reported difficulties in obtaining life insurance, 98 (15%) obtaining mortgage.

Dieng M. et al [47]	Cross sectional survey	Stage 0/I/II Mean T since diagnosis: 7.6 (6.5) yrs.	FACT-M [31] Assessment of QOL-8 dimension scale (AQoL-8D) [61] Fear of cancer recurrence Inventory (FCRI) [62]	183 (89%)	High fear of recurrence was associated with a significant decrease of HRQOL. AQoL8D is an alternative to the FACT-M, more sensitive to changes in psychological health and fear of recurrence and can be used to asses utility based health status.	_

Loquai C. et al [63]	Cross-sectional survey	Stage 0/I/II (81%) Stage III (13%) Stage IV (5%) T since diagnosis 0-2 yrs. (44%) 2-5 yrs. (26%) ≥ 5yrs. (31%)	Distress Thermometer (DT) with Problem List (PL) [64]	734 (71%)	_	52 % reported ≥1 emotional problem Presence of emotional problems, family problems and younger age were strongly associated with higher distress. DT and Pl reliable identify distressed melanoma patients.

**Table 4:** Description of the questionnaires used in the referenced studies.

Instrument	Goals	Cancer specific	Melanoma specific	Survivor-specific	Subscales	Remarks
EORTC QLQ-C30 [28]	Global HRQOL	yes	no	no	5 functional scales: physical, emotional, role, cognitive 9 symptom scales: fatigue, pain, nausea, dyspnea, appetite loss, insomnia, constipation, diarrhea 1 summary scale. 30 items	Possible lack of sensitivity for use in melanoma survivors to evaluate HRQOL [41]. Symptoms not specific for melanoma survivors. Not validated in cancer survivorship or in melanoma patients.

FACT-General [30]	Global HRQOL	yes	no	no	4 functional scales: physical, emotional, social, functional wellbeing. 27 items	Can be completed by the FACT-M scale.

FACT-Melanoma [31]	Global HRQOL	yes	yes	no	3 functional scales: physical, emotional, social, wellbeing. 27 items FACT-G + 24 FACT-M items	Melanoma specific with a specific post-surgery scale. Validated in all stages of melanoma.

Assessment of QLQ-8 [61]	Global HRQOL	yes	no	no	8 dimensions: 3 physical dimensions (independent living, pain, senses) and 5 mental dimensions (mental health, happiness, coping, relationships, self-worth). 35 items	Is sensitive to changes in mental and emotional health. May also be useful to capture the benefit of psychological interventions and to measure their cost effectiveness.

Impact of cancer (IOC) [29]	Global HRQOL	no	no	no	8 scales: physical functioning, vitality, social functioning, general health, bodily pain, physical and emotional role, mental health. 37 items	Adjustment to changes. Measures positive as well as negative impact of cancer. Not validated in cancer survivorship.

Lymphedema and Breast Cancer questionnaire (LBCQ) [54]	Symptoms and signs of lymphedema	yes	no	no	Assessment of 19 signs and symptoms. 59 items	Used in clinical practice to follow up lymphedema. Can be useful in melanoma survivors as lymphedema impacts on HRQOL and wellbeing [38, 44, 45]. Not validated in the melanoma survivorship setting.

PHQ-9 [51]	Depression	no	no	no	Assessment depressive symptoms. 9 items	Screening for depressive symptoms. Widely used in survivorship trails.

General anxiety disorder GAD-7 [52]	Anxiety	no	no	no	Screening for General Anxiety Disorder (GAD). 7 items	It is not yet known that GAD is present in metastatic melanoma survivors. Not validated in cancer survivorship.

Mental Adjustment to Cancer Scale [56]	Adjustment to cancer	yes	no	no	Measures fighting spirit, anxious preoccupations, helplessness and loneliness and fatalism. Updated scale includes also global adjustment to cancer. 40 items	Satisfactory measure of psychosocial outcome during the disease phase. Not validated in cancer survivorship.

Mental Health Inventory [57]	Psychological distress and wellbeing	no	no	no	Assessment of anxiety, depression, behavioral control, positive affect and general distress. Original 38 items, revised version with 18 items.	Allows screening of emotional distress as well as behavioral aspects. Widely used in the field of cancer. Not validated in cancer survivorship.

Fear of cancer recurrence (FCRI) [62]	Fear for cancer recurrence	yes	no	yes	Evaluates severity, triggers, psychological distress, coping strategies, insight and functional impairments. 42 items	Allows evaluating fear of recurrence of disease, which is in particular of interest in metastatic melanoma treated with immunotherapy in view of the high risk of recurrence, however not validated in melanoma setting.

Distress thermometer [64]	Distress	yes	no	no	Five categories: practical, family, physical and emotional problems, spiritual and religious concerns. 35 items	Useful and easy to use screening tool for emotional distress in clinical practice. Reliably identifies distress in melanoma patients [63].
